# The Opening of the Bedford County Hospital

**Published:** 1899-06-24

**Authors:** 


### The Institutional Workshop.

### THE OPENING OF THE BEDFORD COUNTY HOSPITAL.

The county of Bedford is to be congratulated on the admirable hospital which was opened with considerable ceremony last Wednesday by the Duke of Bedford. It is not every town that possesses or can obtain such a site as that on which this new building stands, nor, may we add, is it every day that the managers of a hospital, which after all is not very ancient, have the boldness to launch out upon a scheme for building an entirely new hospital on a new site rather than tinkering up an old one which they already possess. But by the help of this site and as the result of this boldness, Bedford now possesses one of the most charming hospitals in the country.

The first thing that one is struck by on visiting this institution is the roominess of the site on which it stands. Ten acres of land for about ninety patients leaves ample margin, as is seen at once by the position of the buildings—which stand back behind an ample lawn 230 ft. from the road—and by their arrangement in pavilions over 100 ft. apart.

There is a certain monotony in looking at hospital plans, for almost of necessity there is much similarity in the general arrangements of the blocks of which all modern hospitals consist. And so far as the general plan goes the Bedford County Hospital is no exception to the rule. We have a central administration block, the back of which abuts upon a corridor running right and left. Branching out from this corridor at the back are three pavilions for the patients, while at its extreme end on the right is the chapel. Quite separate from the main building to the extreme left is the out-patient department, while to the extreme right is the nursing home. Far away behind, standing in the midst of the garden of the old hospital is a block of isolation wards, and also far away at the back are the old laundry and mortuary, which will continue to be used. So far there is nothing special. It is in the details that the interest lies. Let us take one of the two-storey pavilions. Entering from the main corridor we find ourselves at the bottom of a staircase leading to the upper ward. The first thing we notice is that each pavilion has its own coal store in which the coal is placed by the contractors, by which means not only is all the labour of distributing the coal saved to the institution, but a check is kept upon the exact amount of fuel used in each pavilion. Here also is a lift. Then we come to a room full of separate cupboards for the patients' clothes, and a room for pails and brushes, and only then do we come to the short ventilating corridor by which the wards are cut off from the rest of the hospital. Thus the air of the staircase is quite separated from that in the wards.

On entering the pavilion we find, on the left, a separate ward, on the right a larder and linen-room; further on is the nurses' kitchen or duty-room, and on the opposite side an admirable convalescent or day room, serving both for upstairs and downstairs wards; and then we reach the main ward for 16 patients. At the far end of the ward is the bath-room on one side and the sanitary arrangements on the other, each well "cut off," and between them is an open verandah, to which the patients have access, and where they can sit or lie in the open air. The length of the principal wards is 69 ft., the breadth 27 ft., and the height 13 ft., thus giving an air space per bed of 1,513 cubic feet per patient. The warming is by means of two double Shoreland Faience Stoves, with air inlets and descending flues. Through these stoves warm fresh air is introduced. Hot water pipes also are arranged round the walls, and fresh air inlets are placed at the level of the floor.

The children's ward is, we think, unique. It is certainly the most beautiful children's ward that we have seen. In all the wards the walls have a dado of green glazed bricks, above which in the general wards is Parian cement. But in the children's ward the upper part is entirely lined with tiles, and, between the windows, with painted panels in faience illustrating old nursery rhymes and tales.

Thus we get the great picture difficulty solved. The pictures are part of the walls, and are very good pictures, too. "Little Bo-Peep" and all the rest of our nursery acquaintances stand before us, not as dusty pictures, or dirty cuttings from illustrated papers, but in the form of good vitrified tiles, which can be washed and kept clean. All these, as well as the tiling in the operating-room, are by Messrs. Simpson and Son, London, and are highly creditable to them.

The children's pavilion is of one storey only; the rest have two, the upper one being similar to the lower one, except that it has no convalescent-room,

The operating-room is very well arranged, and is fitted with all modern appliances, having an anæsthetic-room and a surgeon's dressing-room attached.

The nurses' home stands quite separate from the hospital. On its two floors it provides accommodation for eighteen nurses and sisters, each of whom has a separate bedroom—pretty little rooms, which, although all the same in size, vary among each other in the colour of their walls, carpets, &c. This seems a very happy thought as diminishing monotony and taking away from the "institutional" character of the building. Although what some would term an extra, and separate from the ordinary work of a hospital, the chapel is always an important adjunct to such an institution, and no account of the Bedford County Hospital could be considered complete without a reference to its very beautiful chapel, the whole of the interior of which, including the organ, the stained glass, the oak work, and the very handsome wood carving, and even the tiles on the walls, are the gift of the chairman of the hospital, Mr. J. Frederick Nutter.

The cost of the hospital is £38,000, which provides for 90 patients, but the plan is drawn for future extension so as to accommodate about 40 more. The architect is Mr. H. Percy Adams, F.R.I.B.A.; the contractors were Messrs. Kerridge and Shaw, of Cambridge; the carvings in the chapel are by Mr. S. W. Elmes, of London; the hot-water and heating apparatus are by Messrs. Rosser and Russell, of London; many of the ward appliances are by Messrs. Maw, Son, and Thompson; and all the baths, lavatory basins, &c., are of Farnley ware.

